# Effect of Genetic-Environmental Interaction on Chinese Childhood Myopia

**DOI:** 10.1155/2020/6308289

**Published:** 2020-11-07

**Authors:** Xiyan Zhang, Yan Wang, Chenwei Pan, Wenyi Yang, Yao Xiang, Jie Yang, Fengyun Zhang

**Affiliations:** ^1^Department of Child and Adolescent Health Promotion, Jiangsu Provincial Center for Disease Control and Prevention, Jiangsu, China; ^2^Public Health Research Institute of Jiangsu Province, Jiangsu, China; ^3^School of Public Health, Soochow University, Suzhou, China; ^4^School of Public Health, Southeast University, Nanjing, China

## Abstract

**Objective:**

The purpose of this study was to evaluate the effect of genetic-environmental interaction on myopia in Chinese children aged 6 to 9 years.

**Methods:**

Students had the physical examination and were required to provide basic demographic information. Their families were asked to fill in a questionnaire concerning factors related to myopia. Multiple regression analysis was performed, and adjusted risk ratio values were calculated to assess the role between gene and environment. Value of the environmental and genetic index (EGI) was calculated to demonstrate the effect of genetic-environmental interaction on myopia.

**Results:**

The prevalence of myopia maintained at a high level. EGI was calculated as 0.125 suggesting that genetic factors may play the 12.5% role in the formation of myopia and environmental factors may play a role of 87.5% in the formation of myopia.

**Conclusions:**

For young pupils aged 6 to 9 years, myopia prevalence maintained at a high level, and duration of homework time and staring at electronic screen were the strongest factors associated with myopia. The calculated value of EGI was low, which suggests that environmental factors may play the leading role in the formation of myopia. A long-term follow-up research to improve the accuracy value of EGI is our next job.

## 1. Background

As a global public health problem leading to visual impairment and blinding complications [1], myopia brings a lot of personal concern and social burden because of the need for correction for refractive errors to avoid visual damage [2]. In China, the prevalence of myopia, which was reported to be higher than that in other countries [3–8], has markedly increased in the past three decades. Therefore, Chinese government has issued *Implementation Plan of Comprehensive Prevention and Control of Myopia in Children and Adolescents* in 2018 to cope with the serious situation [9]. Located in the eastern China, Jiangsu Province is one of the most developed regions with higher education level [10]. The national survey on students' constitution and health from 2005 to 2014 indicated that Jiangsu Province had the highest prevalence of uncorrected visual acuity (UCVA) among 31 provinces in China [11]. Myopia is a complicated disease, and both genetic and environmental factors contribute to its development [12]. Gene-gene and gene-environment interactions are key features in the development of complex diseases such as myopia. Gene-environment interaction can be defined as “a different effect of an environment exposure on disease risk in persons with different genotypes,” or alternatively “a different effect of genotype on disease risk in persons with different environmental exposures” [13]. An interaction happened when the effect of one factor is only evident in the presence of another, and factor could be genetic factors or environmental factors. However, Data on the effect of the gene-environment interaction to myopia in the literature is rare for young Chinese children. This study is aimed to evaluate the effect of genetic-environmental interaction on myopia in Chinese children aged 6 to 9 years.

## 2. Methods

### 2.1. Study Design and Setting

This study is based on the project “surveillance for common disease and health risk factors among students” in Jiangsu Province, which is conducted during 2018-2019 academic year.

### 2.2. Participants

We randomly selected schools in each 12 urban districts/rural counties in Jiangsu Province, and randomly sampled the students from each school. In each of the selected schools, teachers, students and doctors were included in this study.

### 2.3. Definition of Myopia

Myopia was defined as −0.50 diopters (*D*) in the worse eye, which was defined as the eye with the greater absolute value of refractive error (spherical equivalent) [14].

### 2.4. Data Sources and Ethics Statement

Based on the project “surveillance for common disease and health risk factors among students” in Jiangsu Province, we investigated 2,623 students aged 6 to 9 years and their families in Jiangsu Province. Meanwhile, students' parents were asked to fill in a questionnaire covering basic information such as outdoor activity time and parental spherical equivalent value.

Pupils participated in an ophthalmic examination with autorefractor applied with cycloplegia. The cycloplegic refraction is measured using tropicamide phenylephrine eye drops every 5 min, 3 times, and then refractive error is measured 30 min after the first drop of tropicamide by autorefractor with five repeated measurements.

The inclusion criteria for our subjects were (1) no other serious eye diseases; (2) Chinese Han Nationality students; (3) aged from 6 to 9; (4) ability of parents/guardians to provide informed consent.

The spherical equivalent of the refractive error was calculated as the spherical value of refractive error plus one half of the cylindrical value.

The study protocol was approved by the Institutional Review Board of Ethics Committee of Jiangsu Province, and detailed information can be found in the previous article [15]. Written informed consent was obtained from the child's guardian.

### 2.5. Statistical Analysis and Assessment of Environmental-Genetic Roles

Descriptive statistics are used to summarize the variables regarding the characteristics of students aged 6 to 9 years. Continuous variables such as spherical equivalent (*SE*) were presented as mean with standard deviation (*SD*). Multiple regression analysis was performed and adjusted risk ratio values were computed to assess the relationship between myopia and other risk factors [16–18].

The myopic family group, in which both father and mother suffered from myopia, represents the total effect of genetic susceptibility (*G*) and environmental factors (*E*) : *P*_*d*_ = *G* + *E*. The nonmyopia family group, in which both father and mother were nonmyopic, represents the total effect of environmental factors (*E*) : *P*_*n*_ = *E*. The genetic predisposition (*G*) could be represented as [19]

(1) $$\begin{array}{c} P_{d}-P_{n}=G=\text{EGI}. \end{array}$$

Data were analyzed using SPSS V.20.0 software.

### 2.6. Bias

In this study, we will measure each child's values of refractive error three times by automation optometry instruments. Then, our quality control observers compare three refraction values. If the difference between these three values of refractive error (spherical equivalent) was more than 0.50 diopters (*D*), the students would be required to go back and measure one more time. The observers, who are responsible for measurement, were blinded. They did not know when these students received a repeated measurement.

## 3. Results

A total of 2623 families were included in this study. The prevalence of myopia among young boys and girls maintained at a high level, which showed 18.8% for male students and 16.8% for female students. The average value of spherical equivalent (*SE*) for worse eye was 0.3 ± 1.2D for boys and 0.4 ± 1.2D for girls, respectively. For boys, the proportion of father's and mother's education level (high school or above) was 73.8% and 67.5%, respectively, which were 77.9% and 70.7% among girls. The prevalence of father's and mother's myopia was 33.0% and 36.6%, and 36.5% and 40.1% in boys and girls, respectively (Table 1).

Compared with nonmyopic students, students with myopia had higher proportion and longer time spent in near-work such as doing homework (adjusted RR > 1.0, *P* < 0.001) and watching TV (adjusted RR > 1.0, *P* = 0.031). Myopic students were more likely to have less time for sleep (adjusted RR＜1.0, *P* < 0.05), while nonmyopic students spent more time doing outdoor activities (adjusted RR ＜ 1.0, *P* < 0.05) (Table 2, Supplementary Table S1).

We further assessed the *G* × *E* model variables using multiple regression analysis, the variables of father's diopters, mother's diopters, and maximum and minimum diopters of families were included. Variables of nearwork, sleep, and outdoor exposure were excluded (Table 3).

Table 4 shows the data of children with or without myopia from parents. We exhibited the different distribution of refraction between different groups. EGI was calculated as 0.125 suggesting that genetic factors may play a role of 12.5% in the formation of myopia and environmental factors may play a role of 87.5% in the formation of myopia.

## 4. Discussion

The study was to evaluate the effect of the genetic-environmental interaction on myopia in Chinese children aged 6 to 9 years. A total number of 2623 families were included in this study. Prevalence of myopia among young boys and girls maintained at a high level, which showed 18.8% for male students and 16.8% for female students. Duration of homework time and staring at electronic screen were the strongest factors associated with myopia among young pupils. The calculated value of EGI was 0.125 suggesting that genetic factors may play a role of 12.5% in the formation of myopia and environmental factors may play a role of 87.5% in the formation of myopia.

### 4.1. The Prevalence of Myopia among Young Chinese Children

The prevalence of myopia in 2005, 2010, and 2014 Chinese National Students' Constitution and Health survey indicated that the peak prevalence of myopia has become earlier with age and kept at a high level in children [20]. In 2018, the National Health Commission of the People's Republic of China has reported the prevalence of screening myopia for children from kindergarten (aged 6 years) to high school. The prevalence of myopia was 14.5% for children aged 6 years, 36.0% for primary school students, 71.6% for middle school students, and 81.0% for high school students [21]. Similarly, in this study, the prevalence of myopia among children aged 6 to 9 years was 17.9% (95% CI: 16.4–19.3) in Jiangsu Province and kept at a high level.

### 4.2. Major Environmental Risk Factors Associated with Myopia

Cross-sectional and longitudinal studies on Chinese children had found an association between risk factors such as nearwork/outdoor activities and myopia [22–25]. However, data from younger children in China were relatively limited. In this study, we found that age, parental myopia, nearwork activities (homework, watching TV, and playing PC games), sleep duration, and outdoor activities were related risk factors associated with myopia. The report of National Monitoring of Common Disease and Health Risk Factors among Chinese students indicated that the daily outdoor activity time of Chinese students was very low because of the heavy academic burden [26]. In this situation, nearwork has become a relatively important factor contributing to myopia for young children in China. As myopia genes are common in the eastern Asia population, change of lifestyle should be a major focus in the prevention of myopia [27]. A population-based study showed that refractive error increased by 0.10 *D* per 1-hour increase in sleep after adjusting for potential confounders including sex, age, height, education level, economic status, and physical activity [28]. Duration of sleep could affect the development of myopia in younger children. Major environmental risk factors associated with myopia include extended nearwork, minimal outdoor exposure, and parental myopia [15]. Characteristics of myopia-related environmental data are as follows: (1) the data are obtained in the form of self-reported questionnaire. (2) Real-time measurements of nearwork and outdoor exposure will facilitate more accurate evaluation. (3) Quantitative parameters are lack of global standard.

Also, we found a very poor relationship between myopia and air pollution (PM2.5 (*μ*/m^3^), PM10 (*μ*/m^3^), SO2 (*μ*/m^3^), and NO2 (*μ*/m^3^)) or illumination of blackboard/desk (Supplementary Tables S2 and S3). A few of epidemiological studies on association between air pollution and refractive errors were reported. A recent study had reported that myopia was associated with PM2.5 and NOx which could be an indicator of air pollution [29]. Exposure to air pollution had a weak link with development of myopia, and long-term studies should be implemented [30]. The outdoor light environment had been proved to play an important role in the onset and progression of myopia [31], but data concerning indoor light environment were limited. Therefore, we did not include two factors above in this study.

### 4.3. Model of *G* × *E* Interaction

In general, myopia involving both environmental and genetic factors is an ideal disease model for studying interaction effect. The usual analysis model of *G* × *E* interaction is based on the linear regression of genotypic performance on environmental changes (e.g., classic stability analysis). Linear function could partly explain *G* × *E* interaction variations. Therefore, Li et al. [19] used EGI to calculate the *G* × *E* interaction. The EGI is mainly used to observe the prevalence of such diseases in children from disease-parent groups or one-side disease-parent groups, which is estimated to be rarely exceed 50%. They collected students' and their parents' self-reported education data and visual acuity. The EGI is 0.385, suggesting that genetic factors paly 38.5% in the formation of myopia, and myopia is attributable mainly to nongenetic factors, such as education factors. The uniqueness between two studies is as follows: firstly, previous refractive error values were self-reported. In this study, these values were obtained by automation optometry instruments. Secondly, environment risk factors with statistical significance were added in this study. Thirdly, we focus on students aged 6 to 9 years rather than college students. This eliminates the confounding bias caused by age.

### 4.4. Effect of *G* × *E* Interaction

Enthoven et al. [27] performed a cross-sectional study among children aged 9 years old with a myopia prevalence of 12%. They found that increased nearwork and decreased outdoor exposure in childhood significantly enhance the effect of myopia genes. However, Fan et al. [32] found that there was no indication that variant or GRS effects altered depending on time outdoors. Genes and environment (involving nearwork or time outdoors) interactions were rare or absent for the vast majority of the GWAS-identified SNPs. Limitations for *G* × *E* interaction analysis are as follows: firstly, these results were based on a cross-sectional study, and a long-term follow-up research was needed to determine the *G* × *E* interaction analysis. Secondly, pathological mechanism of *G* × *E* interaction should be further conducted. Thirdly, the accurate of environmental data measurement is enhanced to improve the accuracy of the EGI model.

## 5. Conclusion

For young pupils aged 6 to 9 years old, the prevalence of myopia maintained at a high level, and duration of homework time and staring at electronic screen were the strongest factors associated with myopia. The calculated value of EGI was low, which suggested that environmental factors might play the leading role in the formation of myopia, and a long-term follow-up research to improve the accuracy value of EGI would be our next job.

## Figures and Tables

**Table 1:** Basic profile of children and their parents who received investigation.

Male children (1407)	n/N	Proportion (%)/mean ± SD	Female children (1216)	n/N	Proportion (%)/mean ± SD
Children's prevalence of myopia	265/1407	18.8		204/1216	16.8
Children's mean SE (worse eye, D)	—	0.30 ± 1.20		—	0.40 ± 1.20
Father's prevalence of myopia	465/1407	33.0		444/1216	36.5
Father's mean SE (worse eye, D)	—	−3.40 ± 1.90		—	−3.40 ± 1.80
Mother's prevalence of myopia	515/1407	36.6		488/1216	40.1
Mother's mean SE (worse eye, D)	—	−3.40 ± 1.80		—	−3.40 ± 1.90
Father's education (≥high school level)	1038/1407	73.8		947/1216	77.9
Mother's education (≥high school level)	950/1407	67.5		860/1216	70.7

**Table 2:** *G* × *E* interaction factors of myopia for Chinese children.

Variable	*β*	SE	*P*	Adjusted RR (95% CI)
Age	0.565	0.051	0.000	1.759 (1.591–1.945)
Father's myopia	0.373	0.093	0.000	1.452 (1.209–1.742)
Mother's myopia	0.441	0.092	0.000	1.554 (1.297–1.863)
Homework (2-3 hours)	0.364	0.125	0.004	1.440 (1.127–1.840)
Homework (>3 hours)	0.526	0.229	0.022	1.692 (1.079–2.652)
Watching TV (1-2 hours)	0.399	0.203	0.049	1.490 (1.002–2.217)
Playing PC games (1-2 hours)	−0.428	0.205	0.036	0.652 (0.436–0.973)
Playing PC games (2-3 hours)	0.651	0.326	0.046	1.918 (1.012–3.636)
Sleep duration	−0.122	0.059	0.037	0.885 (0.789–0.993)
Lunch break at school	−0.212	0.104	0.042	0.809 (0.659–0.993)
Outdoor activities at noon	−0.410	0.166	0.013	0.664 (0.480–0.918)

**Table 3:** Multiple regression analysis using 2623 families to assess environmental and genetic model in myopia.

Variable	*β*	SE		95% CI for *β*	*P*	Adjusted *R*^2^
Max SE (D)	−0.54	0.03	−0.297	−0.179	<0.001	0.402
Min SE (D)	−0.929	0.037	−0.248	−0.103	<0.001	
Father SE (D)	0.511	0.028	0.069	0.179	<0.001	
Mother SE (D)	0.665	0.031	0.079	0.201	<0.001	
Homework (2-3 hours)	−0.063	0.101	−0.285	0.113	0.393	Removed
Homework (>3 hours)	0.079	0.246	−0.213	0.76	0.268	Removed
Watching TV (1-2 hours)	−0.091	0.079	−0.256	0.056	0.207	Removed
Playing PC games (1-2 hours)	−0.012	0.065	−0.138	0.117	0.872	Removed
Playing PC games (2-3 hours)	0.08	0.158	−0.136	0.488	0.266	Removed
Sleep duration	−0.097	0.029	−0.101	0.014	0.138	Removed
Lunch break at school	0.076	0.051	−0.04	0.162	0.235	Removed
Outdoor activities at noon	−0.006	0.071	−0.146	0.134	0.931	Removed

**Table 4:** Effect of environmental and genetic factors on myopia occurrence.

Parents with or without myopia	Diopters in children (quartile)			Myopia rate in children	EGI^*∗*^
	25th	50th	75th		
With myopia	−0.50	0.40	0.90	26.3%	0.125
Without myopia	0.00	0.60	1.10	13.8%	
One-side parent with myopia	−0.10	0.50	0.90	18.7%	
*P*	<0.001			<0.001	

^*∗*^EGI comprehensively reflects both the effects of genetic factors and environmental factors.

## Data Availability

All relevant data are shown within the manuscript, but original datasets cannot be shared because of involving students' personal privacy.

## Supplementary Materials

Supplementary Materials Supplementary Table 1: characteristics of children from nonmyopic family. Supplementary Table 2: association between air quality (2017.01∼06) and childhood myopia. Supplementary Table 3: association between illumination and childhood myopia.
