# Supplementary material for: Effect of Genetic-Environmental Interaction on Chinese Childhood Myopia
Source: J Ophthalmol. 2020 Nov 7;2020:6308289. doi: 10.1155/2020/6308289 (PMC7670298; doi:10.1155/2020/6308289)
Supplement: Supplementary Materials — Supplementary Table 1: characteristics of children from nonmyopic family. Supplementary Table 2: association between air quality (2017.01∼06) and childhood myopia. Supplementary Table 3: association between illumination and childhood myopia. [file 6308289.f1.docx]

Supplement Table 1 Characteristics of children from non-myopic family

| Environmental factors | | Children from non-myopic family | | | All children | | |
| --- | --- | --- | --- | --- | --- | --- | --- |
|  |  | Myopia (167)  %/ Mean±SD | Non-myopia (1046)  %/ Mean±SD | P | Myopia (469)  %/ Mean±SD | Non-myopia (2154)  %/ Mean±SD | P |
| Near work | Homework (2-3 hours) | 15.5 | 10.4 | 0.070 | 18.1 | 12.3 | 0.001 |
|  | Homework (＞3 hours) | 5.1 | 3.1 | 0.219 | 4.9 | 2.5 | 0.010 |
|  | Watching TV (1-2 hours) | 34.7 | 20.5 | 0.026 | 28.3 | 19.6 | 0.031 |
|  | Playing PC games (1-2 hours) | 25.0 | 26.0 | 0.878 | 17.7 | 26.5 | 0.019 |
|  | Playing PC games (2-3 hours) | 8.3 | 1.9 | 0.012 | 6.1 | 2.6 | 0.024 |
| Sleep | Sleep duration | 9.6±0.8 | 9.6±0.8 | 0.071 | 9.6±0.8 | 9.7±0.8 | 0.031 |
|  | Lunch break at school | 67.7 | 67.0 | 0.869 | 73.3 | 68.1 | 0.025 |
| Outdoor  activities | Outdoor activities at noon | 76.3 | 81.7 | 0.325 | 71.5 | 80.9 | 0.006 |

Supplement Table2 Association between air quality (2017.01~06) and childhood myopia

|  |  | PM2.5(μ/m^3^) | PM10（μ/m^3^） | SO2（μ/m^3^） | NO2（μ/m^3^） | Composite index | Maximum index |
| --- | --- | --- | --- | --- | --- | --- | --- |
| Myopia（%） | Pearson | 0.09** | 0.10** | 0.09** | 0.02 | 0.09** | 0.09** |
|  | P | 0.007 | 0.003 | 0.005 | 0.512 | 0.007 | 0.007 |
| PM2.5(μ/m3) | Person |  | 0.99** | 0.55** | 0.68** | 0.98** | 1.00** |
|  | P |  | ＜0.001 | ＜0.001 | ＜0.001 | ＜0.001 | ＜0.001 |
| PM10（μ/m3） | Person |  |  | 0.55** | 0.62** | 0.96** | 0.99** |
|  | P |  |  | ＜0.001 | ＜0.001 | ＜0.001 | ＜0.001 |
| SO2（μ/m3） | Person |  |  |  | 0.74** | 0.71** | 0.56** |
|  | P |  |  |  | ＜0.001 | ＜0.001 | ＜0.001 |
| NO2（μ/m3） | Person |  |  |  |  | 0.80** | 0.69** |
|  | P |  |  |  |  | ＜0.001 | ＜0.001 |
| Composite index | Person |  |  |  |  |  | 0.98** |
|  | P |  |  |  |  |  | ＜0.001 |

Supplement Table3 Association between illumination and childhood myopia

| Illumination(lx) |  | myopia |
| --- | --- | --- |
| Average illumination of blackboard | Pearson | .007 |
|  | P | .807 |
| Average illuminance of desk | Pearson | .053 |
|  | P | .055 |
